# Bringing scientific rigor to community-developed programs in Hong Kong

**DOI:** 10.1186/1471-2458-12-1129

**Published:** 2012-12-31

**Authors:** Cecilia S Fabrizio, Malia R Hirschmann, Tai Hing Lam, Teresa Cheung, Irene Pang, Sophia Chan, Sunita M Stewart

**Affiliations:** 1 School of Public Health, The University of Hong Kong, Pokfulam, Hong Kong; 2 Hong Kong Family Welfare Society, Pokfulam, Hong Kong; 3 Caritas, Pokfulam, Hong Kong; 4 School of Nursing, the University of Hong Kong, Pokfulam, Hong Kong; 5 University of Texas Southwestern Medical Center at Dallas, 5323 Harry Hines Boulevard, Dallas, Texas, 75390, USA

**Keywords:** Community interventions, Chinese, Parenting, Community-based participatory research, Randomized controlled trials

## Abstract

**Background:**

This paper describes efforts to generate evidence for community-developed programs to enhance family relationships in the Chinese culture of Hong Kong, within the framework of community-based participatory research (CBPR).

**Methods:**

The CBPR framework was applied to help maximize the development of the intervention and the public health impact of the studies, while enhancing the capabilities of the social service sector partners.

**Results:**

Four academic-community research teams explored the process of designing and implementing randomized controlled trials in the community. In addition to the expected cultural barriers between teams of academics and community practitioners, with their different outlooks, concerns and languages, the team navigated issues in utilizing the principles of CBPR unique to this Chinese culture. Eventually the team developed tools for adaptation, such as an emphasis on building the relationship while respecting role delineation and an iterative process of defining the non-negotiable parameters of research design while maintaining scientific rigor. Lessons learned include the risk of underemphasizing the size of the operational and skills shift between usual agency practices and research studies, the importance of minimizing non-negotiable parameters in implementing rigorous research designs in the community, and the need to view community capacity enhancement as a long term process.

**Conclusions:**

The four pilot studies under the FAMILY Project demonstrated that nuanced design adaptations, such as wait list controls and shorter assessments, better served the needs of the community and led to the successful development and vigorous evaluation of a series of preventive, family-oriented interventions in the Chinese culture of Hong Kong.

## Background

This paper describes the process of developing an evidence base for community-developed programs in Hong Kong. We discuss the issues that arose and the solutions we explored in applying scientific rigor to a series of preventive interventions to enhance family relationships in the Chinese culture of Hong Kong.

While considerable evidence exists for behavioral family interventions that promote positive and consistent parenting practices to enhance the parent–child relationship [1], there is a dearth of evidence for sound paren-ting interventions that are culturally relevant to Chinese cultures. Therefore, when University of Hong Kong, School of Public Health (HKUSPH) was awarded funding by the Hong Kong Jockey Club Charities Trust to test a series of preventive interventions that targeted family relationships, the academic research teams began an exploratory process to develop and evaluate locally developed interventions. The funding was under the auspices of the larger program, FAMILY: a Jockey Club Initiative for a Harmonious Society (the FAMILY Project), which had an overarching goal to build intergenerational family ties to enhance three key outcomes salient to families in Hong Kong, health, happiness and harmony. In addition to the intervention research, the FAMILY Project conducted a cohort study to identify risk factors for impairment in family function and offered social marketing programs to enhance family relationships [2].

In Hong Kong, social service agencies offer a multitude of community-based programs. These agencies, largely funded by the government, provide most programs free of charge or at low cost and serve all segments of society. This differs from other parts of Asia where a fiscally constrained social sector might only serve the neediest. In the interest of building capacity, the funder suggested four non-governmental agencies, as they were all well-established and had many centers throughout the territory. The foundation that funded the FAMILY Project provides significant support for the social service sector in Hong Kong and knew these agencies to be forward-thinking. The four agencies have varied backgrounds and missions: three are faith-based (Caritas–Hong Kong, Sheng Kung Wei Welfare Council and Hong Kong Christian Services) while the other has roots in local welfare benevolence (Hong Kong Family Welfare Services). None of these agencies had previously participated in any rigorous scientific studies of their programs with academic public health partners, as there is little incentive or reward in Hong Kong for ‘research,’ however, their expertise in program provision and their access to diverse communities made them attractive research partners.

## Methods

As discussions with potential community partners deve-loped, the research teams began to consider the adoption of a more comprehensive approach to working together by applying a community-based participatory research (CBPR) framework. While partnerships between academic researchers and community practitioners have been used extensively in the West to address complex psychosocial and health issues, there is little in the literature to guide development of such partnerships in Asia. Israel, Schulz, Parker and Becker [3] define CBPR in public health as a “collaborative approach to research that equitably involves community members, organizational representatives and researchers in all aspects of the research process” (p. 177). By definition, the CBPR approach is ideal for export to nonwestern cultures. Its emphasis on local community norms and approaches and its conceptualization of academics and community members as partners ensures that the direction of projects is locally relevant and acceptable and the philosophy encompasses potential social and cultural issues.

## Results

Four research partnerships developed over the period of the next 18 months and each conducted a randomized controlled intervention trial, results published elsewhere [4,5] (Table 1). Each research team was composed of four to eight members with complementary experience. The community agencies’ partners were typically led by an experienced social worker, supported by two to three other social workers, and administrative staff. The academic members included psychology and public health researchers with community-partnership experience as academic investigators, and a research assistant.

**Figure 1:**
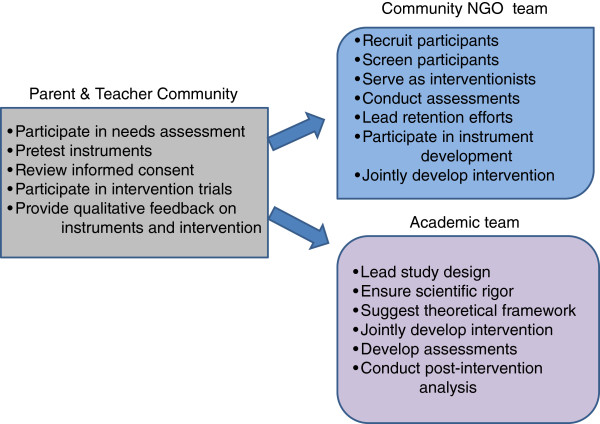
**Illustration of how the parent &teacher community, the NGO, and the academic team impacted the study.**

### Cultural adaptations

While some of the challenges the research teams encountered may be common to other academic-community partnerships due to the two fields’ different outlooks, priorities and languages, the teams’ also encountered challenges that were specific to this Chinese culture. Both types of challenges are reported, as CBPR was new to Hong Kong and often the etiologies of difficulties were difficult to tease out. The largest concerns were that the objective of applying scientific rigor within the CBPR framework would be difficult because Chinese society’s hierarchal nature might hamper an equalitarian partnership and that the cultural propensity to avoid conflict might inhibit resolution of differences in the research design and implementation.

In Chinese society, strong relationships are important and provide a means of surmounting conflict, yet the partners had no prior working experience with each other. The academic team members, particularly those trained in the West, were comfortable with open discussion but needed to find methods of fostering the type of discussion that could lead to problem-solving. Fortunately, due to low personnel turnover, stability of the teams was not a major issue; however, trust and comfort took some time to build. Fortunately, early in the research process the teams undertook an intensive needs assessment process that started the process of building relationships. The needs assessments were designed to understand the target audiences’ priority parenting issues and how they currently addressed them, as well as to obtain feedback on such practical issues as recruiting messages, and optimal time and venue for programs. Each research team conducted four to twelve groups with parents and other stakeholders, which gave the teams the opportunity to work together, experience the same data, and come together to discuss results and make conclusions. This model of a reflective research process was important both for the data it generated and as an important trust-building exercise for the teams’ relationships. In essence it also served as a needs assessment for the teams to understand the community clients served and each others’ work style parameters, and to learn from each others’ unique areas of expertise.

As additional study design and implementation areas were discussed, it became apparent that a decision-making rubric would also be helpful to work through decisions in a culturally appropriate manner. Although HKUSPH had been awarded overall funding for the FAMILY Project, each research-community partnership needed to apply for project-specific funding. It was decided that the agencies would be the applicant and grant recipient and that HKUSPH would assist and endorse the grant-writing process. During the writing process, the teams had collectively negotiated roles and responsibilities based upon their relative areas of expertise. The community-partners would lead recruitment, screening and retention efforts, would staff the administrative needs of the studies, and in most cases would serve as interventionists for the programs. Additional stakeholders, such as the parents and teachers, would participate in a needs assessment and provide feedback on assessment tools, consent forms, and the intervention itself. Concurrently HKUSPH would lead development of the study designs, the interventions’ theoretical frameworks, the assessments and the analysis. In addition, HKUSPH institutionally required that the Principal Investigators (PI) come from the university, in part to lead the request for ethical approval for human subject research (each individual study received approval from the Institutional Review Board (IRBs) of the University of Hong Kong). This delineation drew on each partner’s strengths, in conjunction with the community, but relied upon mutual decision-making in all aspects of the study (Figure 1).

This grant-determined role delineation influenced what came to be an informal decision-making rubric that would aim to reduce potential sources of disagreement in project design and implementation [3]. Once the teams had collectively decided on the RCT design, essential parameters of the scientific design became the framework for what needed to be kept scientifically firm. Eventually this came to include the establishment of separate experimental and control groups, reduction of bias through rigorous randomization procedures, use of manualized interventions, clear protocols for data collection and control and fidelity checks of the intervention. Other areas were open to discussion with probable resolution biased toward accommodation of the realities of the community-setting. The teams adapted the slogan “Best science; best practices” to indicate that the academics’ focus on scientific rigor could be balanced with the agency partners’ expertise in the community.

### Recruiting community agency partners

The academic partners encountered their first challenge in recruiting the community agencies’ participation and acceptance of the RCT design. For the academics, working with the agencies was congruent with the intent for these studies to test implementable intervention programs rather than isolated academic research. It also reinforced the opportunity to enhance community research capacity by reducing barriers to knowledge transfer and program dissemination [6,7]. However, the academic teams were concerned that CBPR’s highly collaborative approach could pose some initial challenges in this Chinese culture where the public and academics interact minimally and the authority of experts is highly valued. From the initial contacts, the academic teams used the framework’s emphasis on co-learning to provide language to reassure the social workers of the value of their local clinical knowledge that they would add to the partnership.

For the four agencies, their decisions to partner were complex. In most communities the agencies run integrated family centers offering a roster of programs from remedial to preventive, however, the reactive nature of their work tend to weigh the bulk of their efforts against diagnostic and treatment programs. For those agencies that sought to offer more strength-based programs for the family, this research opportunity offered incremental resources to extend their services that were not available through other funding channels. In addition, after initial discussion about the evidence-generating process, even the agencies with confidence in their programs’ outcomes began to understand that they had not demonstrated effectiveness quantitatively. Therefore, this funding offered a financially advantageous way to align themselves with the locally-prestigious university and to access resources to create the evidence. However, whereas the academic partners were comfortable that research cannot guarantee positive results, the community partners were concerned that they would invest a considerable amount of time but ultimately the evidence might not support the effectiveness of their programs. The academic teams discussed how success did not have to mean getting the right results, but might better be described as a breadth and depth of feedback on program outcomes. This discussion was coupled with the reassurance of the funder and the university academic leaders, both of whom had long-standing experiences and good relationships with many other agencies, and enabled the agencies to take the leap of faith that testing their programs was not a “pass or fail” endeavor, but a scientific enquiry into overall effect sizes as well as individual causal elements. Eventually the agencies decided that the benefits of the evidence and insights generated, the additional services made available, and the bolstering of their capacities outweighed the risks of potentially demonstrating ineffectiveness.

### Study design

The first area of partner discussion was the study design. The academic partners wanted to apply the highest scientific standard of a randomized controlled trial (RCT) to minimize selection bias and maximize the strength of the evidence generated, given the small to medium effects sizes expected for the studies. The community agencies were eager to learn about this type of study, but cautious about the implications for participants and administration. In exploring alternate study designs, community members struggled to understand the need for the rigors of the RCT design. Recognizing that many agencies and social workers do have uncertainty about the effectiveness and long term outcomes of their interventions, the academic teams used concrete examples to illustrate, such as: if an agency had 100 suicidal people who received the same intensive counseling intervention and within a year five of them committed suicide, did that mean that the program “failed or killed” five people or that it “succeeded with or saved” 95 people? Such a question could not be reliably answered except by an RCT with a control group offering *no* or *less intensive* counseling and the intervention’s effectiveness could only be reliably quantified by the difference in the suicide rate between the two groups. With the resulting effect size estimate, a cost effectiveness analysis could also be conducted. This tangible example was persuasive to the agency partners and helped them become more comfortable with the RCT design.

Almost immediately the research teams encountered a study design element that fell into a gray area of the rubric, as community partners were uncomfortable that those assigned to the control group would be seemingly denied the program. The academic partners had not anticipated this issue as their training supports the ethics of withholding unproven interventions. However, after extensive discussion with the community partners, who are trained to provide assistance to all, the academic teams realized that this issue could be addressed, in addition to the “principle of uncertainty,” with some flexibility without jeopardizing the integrity of the design. Each research team problem-solved ways to alter the designs to satisfy academic and community concerns, without affecting the RCT design [8]. Two of the teams felt so strongly about service provision for all study participants that they decided to add a waitlist control, at their own expense, that offered participants an experimental intervention after the research study was completed. One research team added a family outing as a control group activity and the other team used an attention control (Table 1).

**Table 1:** Overview of community-based interventions

**Name of Study (Community Partner)**	**Transition Point**	**Target Population**	**Study Design**	**Intervention Approaches**	**Outcomes**
***FAMILY: Effective Parenting Programme ***(Caritas, Hong Kong)	Moving into more difficult academic environment	Mothers of children aged 6-9 or P.1-P.3	3-arm design	Arm A: Enhance parents’ mood regulation (adapted from NGO program) Arm B: Enhance positive parenting techniques (new) Arm C: Assessment-only control	Improve parent-child relationship
***FAMILY: Happy Transition to Primary One ***(Hong Kong Sheng Kung Hui Welfare Council)	Entering primary school	Children aged 5-6 (pre-P.1) and their parents	2 x 2 design	(All adapted from NGO program) Arm A: School skills training for children (child only) Arm B: Positive parenting skills(parent only) Arm C: Positive parenting skills + school skills training for children. (parallel training) Arm D: Placebo control – parent-child outing (1 session only)	Improve family relationship, happiness and harmony during the transitional period
***FAMILY: H.O.P.E. Project ***(Hong Kong Christian Service)	Moving into more difficult academics	Parents of children aged 8-10	2-arm design	Arm A: Hope Education Training Program (new) Arm B: Waitlist control group	Enhance *Hope* level; enhance child resilience; improve parent-child relationship
***FAMILY: Harmony@Home ***(Hong Kong Family Welfare Society)	Early adolescence	Parents of children aged 10 -13	3-arm design	Arm A: Existing Alternative to Violence Programme (NGO program, modified for parents) Arm B: Positive parenting (new) Arm C: Waitlist control	Reduce parent-child conflict; improve parent-child relationship

### Program selection and configuration

Program selection was driven by the community partners. The FAMILY Project sought to assist the community partners in creating an evidence base for their choice of programs, within the funder’s parameters for preventive family programs. Here the academics worked closely with their community partners to optimize their programs by clarifying program objectives, diagramming behavior change pathways, and focusing content. Social service agencies in Hong Kong typically offer programs ranging from five to ten sessions, or perhaps a weekend-long program, that included many topics and methods under one umbrella topic, so the community partners were reluctant to limit the length and content of the intervention programs. Previous programs were typically offered to only a small number of participants, and as their data from post-program customer satisfaction-type surveys indicated that the program content was well received by this self-selected group, the agencies were not concerned that lengthy program configurations might limit enrollment. The academic partners acknowledged that the effect size might be greater for more intensive programs but were more concerned that the lengthy programs’ significant cost and time commitments might not only limit broader reach but might also inhibit longer-term sustainability of the programs. Understanding the implications of this issue to implementation and resources, the academic teams suggested a data-driven approach resolution rather than authority or expertise-driven decisions. Needs assessment groups had not yet been conducted so their discussion guides were broadened to include queries about schedule feasibility. When the assessments were conducted among the relatively “healthy” target populations drawn from a universal, non-restricted population, the needs assessments found that most potential participants preferred programs that were short in length and duration. In addition, the community partners sought advice from other social workers with experience in the agencies’ programs, who concurred that the programs could be shortened by aiming at more limited but focused and measurable outcomes. This needs assessment data, supplemented with peer approval, was convincing for all partners. Therefore, the programs were restructured to be shorter and more convenient for the target populations.

Here again the use of a locally relevant example helped drive the intervention design. Chinese *24 herb tea* was utilized as a tangible example to model the need to focus program content within the shortened program. This tea is widely perceived as an effective prevention or treatment for a variety of mild ailments, yet no one has tested which of the 24 herbs is the causal component. Likewise the agencies’ lengthy programs with multiple components needed to isolate what they perceived to be the most effective components, which then could be tested more easily by RCTs.

### Assessment

Assessments were another area where community needs drove adaptation to the research design. Academic researchers often utilize lengthy validated tools and third party observations in laboratory studies. However the agencies brought forward concerns about the community’s willingness and ability to submit to lengthy assessments and to recruit significant others to provide observations of behavior change. This reticence might be common in many community settings, but was particularly a barrier in this society that values the primacy of the family and is reluctant to share personal issues with non-family members. The research teams accepted these concerns and further exploration illuminated multiple ways to measure success of a program, such as shorter quantitative assessments supplemented with qualitative assessments. Assessment timing was also adapted to minimize the burden on participants by scheduling assessments concurrent with program boosters or other events.

### Methodological issues

Methodological issues were anticipated to be problematic as academic partners knew that, despite their community counterparts’ experience in recruiting, conducting programs, managing data and retaining the participants for their usual programs, the efforts would need to be more methodical and detailed for RCTs. The concern was how to heighten awareness of the risk of underestimating the volume and change in work between programs delivered as part of a research study compared to programs delivered in the agencies’ usual practice. Unfortunately, some of the academic partners were not successful in this communication, with severe implications for the community partners’ operations and resource capacities. For example the academic partners were not successful in communicating the shift for facilitators, as the research study required an increase both in preparation time and in formalization of intervention delivery. In their usual practice the social workers would develop the outline of a program prior to beginning the first session and then would prepare in more detail before each subsequent session. The actual program delivered might vary from one social worker to the next, or even one social worker might alter it over time. For the research studies, the intervention sessions were manualized to enhance standardized service delivery and quality, and to help ensure that results were likely to be a function of the intervention, not individual differences of the interventionists [8]. The interventionists were expected to devote six to eight hours to each session, which included time to rehearse and prepare before the session, to write notes on the participants after the session as part of their preparation for the next meeting, to rate their own fidelity (and be rated by an observer) to the program manual, and to conduct make-up sessions with those who missed a session. This increase in preparation time and formalization of the intervention delivery were not easy adaptations for the social workers, particularly those brought onto the project on a part-time basis from other pressing responsibilities. Not all facilitators were able to attend the regularly scheduled fidelity and feedback meetings, and early fidelity ratings indicated adherence issues. By this time, the teams had adopted non-judgmental, data-based, continuous quality control efforts, so this was recognized and addressed early.

In some areas, the academic teams were successful in anticipating and training the community partners for the demands of the research studies, such as for recruitment. Typically Hong Kong social service program recruitment was relatively passive, utilizing center-based promotion and announcements to previous service users. However these studies required 100 to 150 participants, depending on the number of experimental arms, to achieve a minimum power for pilot studies. Initially, these numbers were thought to be easy but the reality of slow recruitment surfaced quickly. Therefore the community partners needed to develop new outreach skills and expend significantly more effort for recruiting. Working together, the teams developed promotion plans that involved networking in new ways with other organizations, such as special interest centers, and aggressively followed up with potential participants in order to achieve the target sample size. In post-intervention evaluations, the agency partners reported that they were pleased to have learned new outreach skills and made important new contacts in their communities.

## Discussion

Our experience of utilizing the CBPR framework to enable the process of using RCTs to develop an evidence base for community programs offers the following lessons:

### Underemphasizing the size of the operational and skills shift between usual agency practices and research studies may derail the project

In our studies, the community agencies underestimated the gap between their usual program practices and the administrative needs of a vigorous trial. The unfamiliar study demands, such as recruitment, data management, and retention, were preceded by funding constraints that created issues for hiring personnel. All the projects were funded for 12 – 15 months, so experienced social workers were understandably reluctant to leave a current job for a new job with such a short timeframe. The agencies solved this resource constraint by subventing current social workers from their regular jobs for the study period, filling in with partial positions from other projects, and if necessary hiring less experienced people to fill in for the more experienced social workers. While this process may be familiar to researchers familiar with study grants, this process was unfamiliar to the community partners, who typically receive continuous government funding for intact programs and people. Later in the studies when the operational demands became overwhelming to the funded staff, the community partners addressed these issues on their own and added resources, at their own expense, diverted from other agency work, before they brought the issues up in team meetings. The academic partners were somewhat surprised to learn of the operational difficulties at this later stage, and found that this represented a missed opportunity to share resources, and a perhaps unnecessary exacerbation of the perception of the burden of research.

### Minimizing non-negotiable parameters is essential in implementing rigorous research designs in the community

Because of the different reference points of academic and community partners, we found ourselves in potential disagreement about a number of research design issues. The community partners found it difficult to differentiate the essential design components from those elements that were simply desirable while the academics experienced a similar process of discovery regarding the community agency's outlook. With discussion and purposeful adaptations, such as accommodating control group access to the intervention after the study and limiting the burden of assessments, study feasibility was enhanced for the community partners, without substantial compromises to the scientific rigor.

### Community capacity enhancement is a process

The FAMILY Project’s five-year, multi-study framework provided support and perspective for movement along a continuum of capacity building. As demonstrated in these four pilot studies, the process of developing and evaluating their programs presented opportunities for the academic team to provide their community partners with tools for future work. Capacity growth came through shared experience as well as the provision of tools, such as program development metrics (identification of program objectives and outcomes, and diagrams of hypothesized causal pathways) or program implementation guidance (recruiting marketing plans or data management protocols). At the end of the pilot studies, two of the community partners chose to continue with the FAMILY Project, and were awarded new funding for much larger studies (300 participants), longer follow-up, and a more rigorous control group. They plan to disseminate their work, and have spoken in local conferences about the evidence-generating process. The actualization of “best science; best practice” has evolved significantly from the early days of education about basic elements of scientific rigor to dissemination of experiences and evidence for future endeavors.

## Conclusions

Community programs can and should be tested with scientific rigor. This paper demonstrates the adaptability and value of the CBPR framework in a nonwestern society, to accommodate and advance the effort to create an evidence base for locally developed programs, in part due to the framework’s emphasis on local community norms and priorities. Green and Mercer [7] describe the ultimate benefit of CBPR as a deeper understanding of the unique circumstances and a more accurate framework for testing and adapting best practices to the community’s needs.

The research teams encountered cultural challenges in adapting that surpassed the expected challenges in being the first to apply a CBPR framework to conduct four pilot RCTs in this Chinese society. The research teams anticipated the issues arising from the academics and community practitioners’ different outlooks, priorities and languages. Additionally the teams foresaw that the objective of applying scientific rigor within the CBPR framework would be difficult because Chinese society’s hierarchal nature might hamper an equalitarian partnership and the cultural propensity to avoid conflict might inhibit resolution of differences in the research design and implementation. Initially the teams prioritized relationship development and utilized early developmental research to begin this process. Later the teams developed a decision rubric for design issues that helped preclude conflicts for design issues, in a way that was respectful to the scientific process and to the community partners’ expertise, experiences, needs and concerns.

The process involved growth on both sides of the academic-community partnerships that might be illustrative for other CBPR partnerships. For the community partners, despite the rigid parameters of the RCT design, and the burdensome demands of the research study operations, the teams focused on the desire to generate the best evidence for their programs. In post-intervention feedback, program leaders emphasized that their desire to create evidence for their program was the driving force through the stress and complications of implementing the research. For the academic partners, openness to the community partners’ suggestions outside of non-negotiable parameters to retain scientific rigor led to realistic modifications to the studies’ design and execution that benefitted reach, implementation, and feasibility. The four pilot studies under the FAMILY Project demonstrated that nuanced design adaptations that better served the needs of the community and the partnership teams led to the successful development and evaluation of a series of preventive, family-oriented interventions in the Chinese culture of Hong Kong.

The relationships established have extended beyond the studies’ completion, as community partners continue to work with the academic teams to disseminate the studies and teams stay in contact for consultation on other projects that might benefit from the other partner.

We hope that this learning will help guide development of other efforts to create evidence for community generated partnerships in different cultures, as even the highest standards of rigorous design can be executed with appropriate adaptations. We can finally say, “It can be done”.

## Competing interests

The authors declare that they have no competing interests.

## Authors’ contributions

CF took the lead role in conceptualizing and drafting this manuscript. MH is the PI for one of the studies and contributed to the drafting of the manuscript. SC is co-Principal Investigator of the Family Project and contributed to drafting the manuscript. THL is the Principal Investigator of the Family Project and helped conceptualize and draft the manuscript. IP and TC were the community leaders of two of the studies and they contributed to conceptualizing and drafting the manuscript. SS is the lead Principal Investigator of the five intervention studies, and contributed to conceptualizing and drafting this manuscript. She is the corresponding author of this study. All authors read and approved the final manuscript. The authors declare that they have no competing interests.

## Authors’ information

This research was supported by grants from the Hong Kong Jockey Club Charities Trust. Special thanks to the FAMILY Project team and our community-partners: Caritas-Hong Kong, Hong Kong Family Welfare Society, the Hong Kong Sheng Kung Hui Welfare Council, and Hong Kong Christian Service.

## Pre-publication history

The pre-publication history for this paper can be accessed here:

http://www.biomedcentral.com/1471-2458/12/1129/prepub
